# The New School Absentees Reporting System for Pandemic Influenza A/H1N1 2009 Infection in Japan

**DOI:** 10.1371/journal.pone.0030639

**Published:** 2012-02-17

**Authors:** Takeshi Suzue, Yoichi Hoshikawa, Shuzo Nishihara, Ai Fujikawa, Nobuyuki Miyatake, Noriko Sakano, Takeshi Yoda, Akira Yoshioka, Tomohiro Hirao

**Affiliations:** 1 Department of Public Health, Faculty of Medicine, Kagawa University, Kagawa, Japan; 2 Department of Health and Welfare, Faculty of Medicine, Kagawa Prefectural Government, Kagawa, Japan; 3 Takamatsu City Public Health Center, Kagawa, Japan; 4 Department of Hygiene, Faculty of Medicine, Kagawa University, Kagawa, Japan; Duke-NUS Graduate Medical School, Singapore

## Abstract

**Objective:**

To evaluate the new Japanese School Absentees Reporting System for Infectious Disease (SARSID) for pandemic influenza A/H1N1 2009 infection in comparison with the National epidemiological Surveillance of Infectious Disease (NESID).

**Methods:**

We used data of 53,223 students (97.7%) in Takamatsu city Japan. Data regarding school absentees in SARSID was compared with that in NESID from Oct 13, 2009 to Jan 12, 2010.

**Results:**

Similar trends were observed both in SARSID and NESID. However, the epidemic trend for influenza in SARSID was thought to be more sensitive than that in NESID.

**Conclusion:**

The epidemic trend for influenza among school-aged children could be easily and rapidly assessed by SARSID compared to NESID. SARSID might be useful for detecting the epidemic trend of influenza.

## Introduction

Influenza is one of the most important infectious diseases requiring monitoring and surveillance all over the world [1]. In 2009, influenza A/N1H1 has become public health challenge in the world [2]. In Japan, the first patient with influenza A/H1N1 was reported in May 2009, and the number of patients with influenza A/H1N1 has dramatically increased. It reached the peak in November, and decreased in December 2009 [3]. Influenza pandemic preparedness and control programs have focused on vaccine development and antiviral drugs, which are only partially effective and not always available to all persons at risk [4], [5]. In Japan, National Epidemiological Surveillance of Infectious Diseases (NESID) was launched in 1981. Numbers of patients with influenza who visited the sentinel clinics or hospitals in a week are reported to the government and published as Infectious Disease Weekly Report. However, the process takes almost 2 weeks and it is a disadvantage of the system [6], [7]. In 2009, School Absentees Reporting System for Infectious Disease (SARSID) has been newly developed, and applied for several areas in Japan including Takamatsu city. In Takamatsu City, the first case of pandemic influenza A/H1N1 was reported in June 2009 and the number of patients increased at the beginning of October. SARSID started from October 13, 2009 in Takamatsu City.

In this study, we compared the SARSID with the NESID on pandemic influenza A/H1N1 2009 in Takamatsu city, evaluating the day of the start, the peak and the end of the pandemic. We analyzed the data from October 13, 2009 to January 12, 2010.

## Methods

### Study area

Takamatsu city, Kagawa prefecture, Japan, is located on the northern shore of Shikoku Island (in a temperate zone area). The population of 426,465 people is situated on the Takamatsu city. Currently several public offices of Shikoku district are located in Takamatsu city. Although the surrounds of Takamatsu had been used primarily as paddy fields for agriculture, recently they have undergone rapid changes, developing into residential and/or commercial areas.

### Data Source

The NESID has been the sole surveillance system for influenza in Japan since 1981. After the legislation of “the Law Concerning the Prevention of Infectious Diseases and Medical Care” in 1999, approximately 5,000 sentinel clinics and hospitals participated in the program throughout the country. In Takamatsu city there were 15 sentinel facilities. Sentinel facilities were recruited on a voluntary basis to report the number of cases of influenza for five working days per week to public health centers. Data were forwarded to the local government (prefecture) and the central government (Ministry of Health, Labour and Welfare). Both analyzed the collected data in local and national levels [6], [7].

The SARSID has been newly developed in 2009. At each school, the number of absentees with flu-like symptoms is recorded every morning by homeroom teachers. The class data are forwarded to the school nurse office or school's administrative offices, and inputted into the local database. Data are automatically transmitted to a central database at SARSID. In Takamatsu city, SARSID has been available from Oct 13, 2009.

In this study we compared these two surveillance systems. We obtained daily aggregated data of both databases from Oct 13, 2009 to Jan 12, 2010 through public health center and local education committee with permissions.

### Subjects

All 149 schools of Takamatsu city (51 kindergartens, 50 elementary schools, 27 junior high schools, 17 high schools and 4 special needs schools,) and 54,483 students were participated in SARSID program. Of these 149 schools, 142 schools (51 kindergartens, 50 elementary schools, 27 junior high schools and 14 high schools) and 53,223 students (97.7%) aged 3–18 years were employed as study subjects (**Table 1**). Schools or classes which corresponded to evening high schools, special needs schools, or special needs classes were excluded from the analysis.

**Table 1:** Number of schools (kindergarten, elementary school, junior high school, high school), classes, and students in Takamatsu city.

	All			Subject		
	School	Class	Student	School	Class	Student
Kindergarten	51	277	6,722	51	268	6,634
Elementary school	50	865	24,119	50	748	23,778
Junior high school	27	383	12,033	27	334	11,910
High school	17	333	11,118	14	311	10,901
Other school	4	126	491	0	0	0
total	149	1,984	54,483	142	1,661	53,223

### Indicators

The start, the peak and the end of the pandemic were compared between NESID and SARSID. The start and the end of pandemic in SARSID were defined as the day the proportion of absentees was over or below 2%, which denoted the twice of nonprevalent period. In NESID, the start and the end of pandemic were defined as the day the average of weekly patients per sentinel facilities was over or below 10, which denoted the warning level that government released the information to the public [7].

The peak of pandemic in SARSID and NESID were defined as the day the number of absentees was maximum and the day the average weekly patients per sentinel facilities was maximum respectively. Age specific analyses were also applied for kindergarten; aged 3–6 years, elementary school; aged 7–12 years, junior high school; aged 13–15 years, and high school; aged 16–18 years.

### Statistical analysis

Joinpoint regression program version 3.4.3 (http://srab.cancer.gov/joinpoint/) was used to investigate whether the point of change was different in NESID and SARSID. And we further analyzed about the parallelism and the coincidence: *p*<0.05 was considered to be statistically significant.

The study was approved by the Ethical Committee of Faculty of Medicine, Kagawa University, Japan (H22-2).

## Results

The average rate of absentees was 3.6±1.2% (mean ± SD), and the peak was 5.1% of SARSID in this observation period. The average number of daily patients per sentinel facilities was 5.7±3.2 (mean ± SD), and the peak was 10.0 of NESID in this observation period.

**Figure 1:**
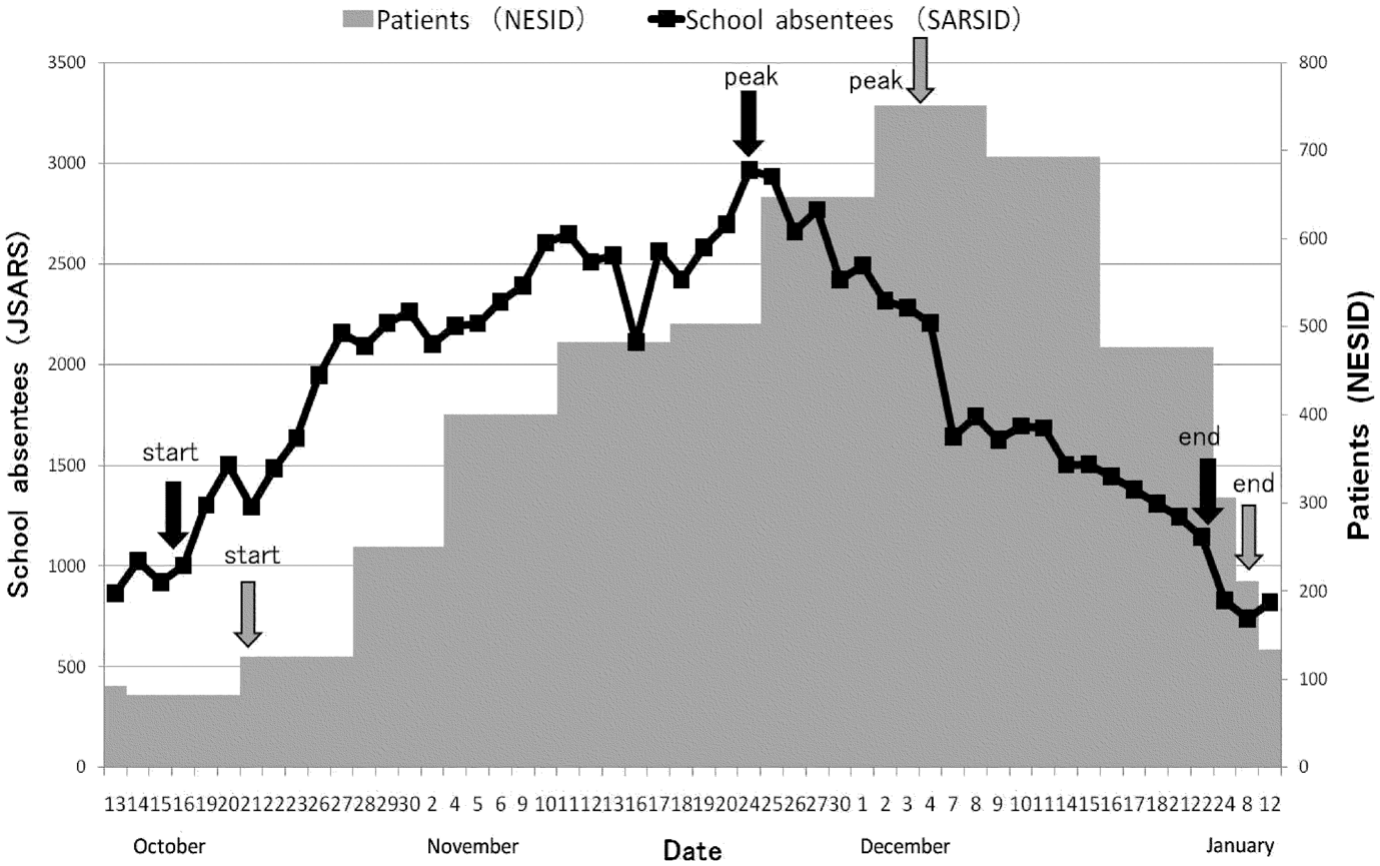
School absentees in SARSID and patients in NESID in Takamatsu city from Oct 13, 2009 to Jan 12, 2010 (all school; aged 3–18 years).

Similar trends were observed in SARSID and NESID. Changes in number of patients of NESID and school absentees of SARSID are summarized in **Figure 1**. The patients began to increase from the end of October, and peaked the beginning of December, then decreased rapidly after the peak. In SARSID, the start of pandemic was October 16, 5 days earlier than NESID; the peak was November 24, 10 days earlier; and the end was December 22, 17 days earlier. In kindergartens, aged 3–6 years old, the start was 8 days earlier, the peak was 16 days earlier, and the end was 17 days earlier. In elementary schools, aged 7–12 years old, the start was 2 days earlier, the peak was 9 days earlier, and the end was 17 days earlier. In junior high schools, aged 13–15 years, the start was 7 days earlier, the peak was 31 days earlier, and the end was 29 days earlier. In high schools, aged 16–18 years, the start was 5 days later, the peak was same day, and the end was 21 days earlier. The start, peak and end days in each school category were showed in **Table 2**.

**Table 2:** Date of increase, peak, decrease in SARSID and NESID, at kindergarten, elementary school, junior high school, high school, and total.

	SARSID			NESID		
	Start	Peak	End	Start	Peak	End
Kindergarten	10/13/09	11/25/09	12/22/09	10/21/09	12/11/09	1/8/10
Elementary school	10/19/09	11/25/09	12/22/09	10/21/09	12/4/09	1/8/10
Junior high school	10/14/09	10/27/09	12/10/09	10/21/09	11/27/09	1/8/10
High school	10/26/09	11/25/09	12/18/09	10/21/09	11/25/09	1/8/10
Total	10/16/09	11/24/09	12/22/09	10/21/09	12/4/09	1/8/10

We analyzed whether the point of change was different between NESID and SARSID using joinpoint regression program. The point of change in SARSID was November 19, 2009, in turn that in NESID was December 5, 2009. We further analyzed the parallelism and the coincidence of these lines. The parallelism was rejected (*p*<0.001), and the coincidence was also rejected (*p*<0.001). Therefore, the point of change was different each other and there were no parallelism and coincidence.

## Discussion

Influenza is one of the most important infectious diseases requiring monitoring and surveillance all over the world. In many industrialized countries, a surveillance system has been executed for detecting influenza. In England and Wales, syndromic surveillance based on National Health Service (NHS) data was reported [8]. In Taiwan, the system that use the data of nationwide emergency department-based syndromic surveillance system was also reported [9]. In USA, the system that obtain data automatically from ambulatory-care encounter records [10] and Emergency Medical Services (EMS) ambulance dispatch records were reported [11]. Ohkusa *et al* reported the system that obtain data from sales of over-the-counter medications [12], [13]. However, these systems have some disadvantages *i.e.* accuracy and rapidity. In pandemic influenza A/H1N1 2009, infection expanded explosively. Early warning is one of the most important arms as preventive measures. Therefore, more accurate and more rapid system has been urgently required.

The method that use school absence data was reported for early outbreak detection study in the United Kingdom [14] and USA [15]. In Japan, SARSID for Influenza has been newly developed in 2009. This system might have advantages in accuracy and rapidity comparing previous systems for the young people aged 3–18 years old. We evaluated the effectiveness of SARSID on pandemic influenza A/H1N1 2009 and compared with NESID in Takamatsu city, Japan. The epidemic trend for influenza among school-aged children could be easily and rapidly assessed by SARSID compared to NESID. To take advantage of SARSID, policies and rules of the effective use of SARSID data are necessary. As for the end of pandemic, school break in December might artificially truncate school absences in SARSID.

We also evaluated the trends of the pandemic in each school category or age group. In all school categories except high school the start day of the pandemic was detected earlier than NESID. Especially in kindergarten and junior high school the start day was detected almost one week ahead of NESID; on October 13 and October 14 respectively. The reason was not clear but the difference of geographical movement might be a major cause. It was commonly considered that the geographical range of daily activities of high school students is wider than that of the students of kindergarten, elementary school and junior high school. In pandemic influenza A/H1N1 2009, activities in daily life might have been related to the morbidity rate of influenza.

Potential limitations still remain in this study. First, school absentees might not be reflect the actual number of patients of pandemic influenza A/H1N1. And the absence rate was not equal to the morbidity of influenza. On the other hand, NESID is not complete surveillance system, so the morbidity could not be calculated in NESID. Second, study area was the specific region and limited observation period. Third, although vaccine inoculation was started from December 25, 2009 in Takamatsu city, the vaccine inoculations to the pandemic influenza A/H1N1 2009 might modify the results of this study.

This study suggested the usefulness of SARSID in influenza prevention and the possibility of infectious disease prevention, however, further studies are required in several areas, several periods, and several infectious diseases.
